# Molecular pathogenesis of hepatocellular carcinoma and impact of therapeutic advances

**DOI:** 10.12688/f1000research.6946.1

**Published:** 2016-05-12

**Authors:** Renumathy Dhanasekaran, Salome Bandoh, Lewis R. Roberts

**Affiliations:** 1 Division of Gastroenterology and Hepatology, Stanford University, Stanford, CA, USA; 2 Department of Medicine, Korle-Bu Teaching Hospital, Accra, Ghana; 3 Division of Gastroenterology and Hepatology, Mayo Clinic College of Medicine, Rochester, MN, USA

**Keywords:** hepatocellular carcinoma, liver cancer, HCC

## Abstract

Hepatocellular carcinoma (HCC) is a leading cause of cancer mortality and has an increasing incidence worldwide. HCC can be induced by multiple etiologies, is influenced by many risk factors, and has a complex pathogenesis. Furthermore, HCCs exhibit substantial heterogeneity, which compounds the difficulties in developing effective therapies against this highly lethal cancer. With advances in cancer biology and molecular and genetic profiling, a number of different mechanisms involved in the development and progression of HCC have been identified. Despite the advances in this area, the molecular pathogenesis of hepatocellular carcinoma is still not completely understood. This review aims to elaborate our current understanding of the most relevant genetic alterations and molecular pathways involved in the development and progression of HCC, and anticipate the potential impact of future advances on therapeutic drug development.

## Introduction

Hepatocellular carcinoma (HCC) is the most common primary liver malignancy and the sixth most common cancer worldwide ^1^. It is an aggressive malignancy with a poor prognosis and is currently the second most common cause of cancer-related mortality. Although more than 80% of the estimated 782,000 new cases of HCC in 2012 occurred in less developed regions of the world, its incidence is increasing worldwide, including in more developed countries ^1^.

The most common risk factors for HCC development are chronic hepatitis B virus (HBV) and hepatitis C virus (HCV) infections, and the prevalence of HCC mirrors the occurrence of these infections ^2, 3^. Other major risk factors include alcoholic cirrhosis, non-alcoholic steatohepatitis (NASH), consumption of aflatoxin-contaminated foods, and exposure to other chemical carcinogens ^4^. Heavy alcohol use increases the risk for HCC and also has been reported to have synergistic effects with other risk factors such as obesity and viral hepatitis ^5, 6^. NASH is the most rapidly growing indication for liver transplantation for patients with HCC in the US, and the annual incidence of HCC in patients with NASH cirrhosis is 2.6% ^7, 8^. There is more recent recognition that the metabolic syndrome and its components such as diabetes and obesity also increase the risk for HCC ^9– 11^. Pre-existing metabolic syndrome has been shown to confer a 2.1-fold increased risk for HCC which is independent of other risk factors ^12^. In this review, we describe the pathogenic mechanisms by which these diverse etiologic factors interact with the molecular milieu in the liver to drive the oncogenesis of HCC.

## Natural history of precancerous lesions

**Figure 1.**
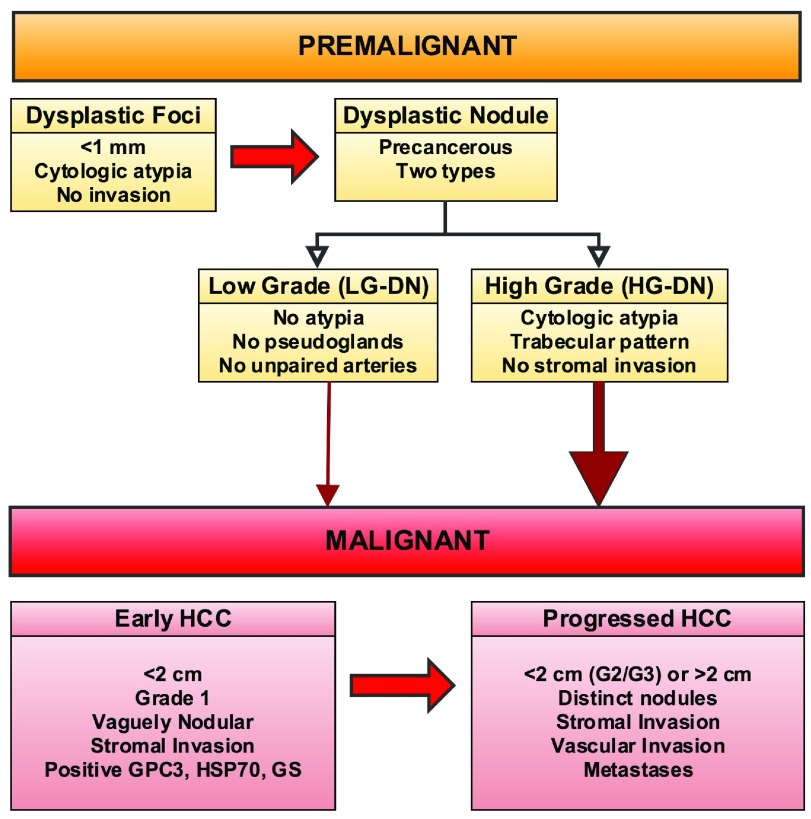
Natural history of precancerous lesions. Dysplastic foci (<1 mm) or dysplastic nodules (DNs) (≥1 mm) are considered precancerous lesions and are classified into low and high grade. Both low- and high-grade nodules have the potential to evolve into HCC, but high-grade DN have a much greater risk. Early HCCs are characterized by the presence of stromal invasion. HCC is further subclassified into early HCC and progressed HCC based on features noted in the figure. Abbreviations: HCC, hepatocellular carcinoma; HG-DN, high-grade dysplastic nodule; LG-DN, low-grade dysplastic nodule.

Most causes of HCC mediate liver injury through the development of liver inflammation and fibrosis, which eventually results in the disordered liver architecture characteristic of liver cirrhosis; thus, cirrhosis precedes HCC in 80–90% of patients. Cirrhotic livers exhibit focal areas of abnormal, immature hepatocytes and these dysplastic foci (<1 mm) or dysplastic nodules (DNs) (≥1 mm) arising in the background of cirrhosis are considered precancerous lesions. DNs are classified into low and high grade on the basis of the presence of atypia and other morphologic features. Although both low- and high-grade nodules have the potential to evolve into HCC, high-grade DN has a much greater risk. Differentiating DNs, especially high-grade DN, from early HCC can be challenging, and an international consensus guideline provides recommendations for making this distinction ^13^. The presence of stromal invasion is considered to be the hallmark feature that differentiates early HCC from DNs. HCC is also subclassified into early HCC and progressed HCC, with differing long-term clinical outcomes ^13^. Early HCC refers to small (<2 cm) well-differentiated (grade 1) tumor nodules with indistinct margins and this is now accepted as a separate entity with a good prognosis. Figure 1 depicts the proposed natural history and typical features of precancerous lesions and HCC.

## Histologic classification of hepatocellular carcinoma

HCC is clinically heterogeneous and interestingly the histopathologic appearance of HCC also exhibits significant heterogeneity. The range of cellular differentiation extends from very well-differentiated to poorly differentiated tumors. HCCs also exhibit varied morphologic subtypes, including biphenotypic HCCs with combined features of hepatocellular and cholangiocarcinoma (4–5%), cirrhotomimetic HCC, clear cell HCC, fibrolamellar HCC, granulocyte colony-stimulating factor HCC with major neutrophilic infiltrates, lymphocyte-rich HCC, myxoid HCC, sarcomatoid HCC, scirrhous HCC, and steatohepatitic HCC. Despite the obvious histological diversity of HCCs, there has been only limited correlation of histological features with known molecular or genetic aberrations in HCC and almost no developed capability to translate histological or molecular characteristics to the selection of optimal therapies. Contemporary efforts to correlate histological subtypes of HCC with molecular features are beginning to yield fruit and improve our understanding of HCC pathogenesis and genotype-phenotype correlations. The recent discovery that a chimeric fusion protein involving protein kinase A is present in 100% (15 out of 15) of fibrolamellar HCCs is one such example ^14, 15^. The chromophobe morphologic subtype of HCC has also been reported to exhibit alternative lengthening of telomeres (ALT), a specific molecular mechanism to overcome replicative senescence ^16^.

## Genetic and epigenetic changes in the molecular carcinogenesis of hepatocellular carcinoma

New advances in next-generation sequencing have yielded significant insights into the genomic landscape of HCC. Several recent studies have explored various aspects of HCC by using whole genome sequencing, whole exome sequencing, RNA sequencing, and genome-wide methylation assays. Results thus far describe a complex and heterogeneous malignancy exhibiting a wide array of genetic and epigenetic changes. Below, we discuss some of the pertinent alterations that play a role in the pathogenesis of HCC.

### Gene mutations

Recurrent somatic mutations in specific genes are well recognized as potential drivers of carcinogenesis. Melanoma and lung cancer have the highest rate of mutations per genome, whereas HCCs usually have an intermediate number of mutations per genome similar to other solid tumors (typically 20–100 mutations per genome) ^17^. The underlying etiology of liver cancer also appears to influence the occurrence of specific mutations. For example, HBV is associated with a relatively high frequency of mutations as it replicates through RNA-mediated reverse transcription, and the HBV reverse transcriptase (HBV RT) does not have proofreading function. In contrast, HCV is a single-stranded non-retroviral RNA virus that, unlike HBV, does not integrate into the host genome. However, HCV can cause double-stranded DNA breaks and increase the mutation frequency. HCV-infected cells exhibit an increased mutation frequency in genes such as the immunoglobulin genes, BCL-6, TP53, and β-catenin (CTNNB1) ^18^. Most recently, HCCs have been observed that have a hypermutated genotype with a mutational spectrum characteristic of that caused by the herbal mutagen aristolochic acid, the toxic ingredient of the Chinese herbal preparation wild ginger ^19^. Below, we discuss some of the commonly observed somatic mutations in HCC.

***Telomerase promoter mutations.*** Telomeres are located at the tips of linear chromosomes and function to protect the chromosome from end-to-end fusion and destruction by nucleases or ligases or both. Telomerase is an enzymatic protein complex made up of the telomerase reverse transcriptase (TERT) and the telomerase RNA component (TERC). Telomerase maintains telomere length by synthesizing specific telomeric DNA sequences and adding them to the end of the chromosome. Telomerase expression is usually suppressed in mature adult cells. Thus, DNA polymerase is unable to fully replicate the terminal chromosomal segment and telomeres become progressively shorter with repeated cell divisions. In chronic liver injury where there is high cell turnover, telomere shortening is accentuated. Telomere shortening beyond a certain critical length leads to activation of a DNA damage program which results in apoptosis or cellular senescence that results in the inability of the liver to fully regenerate a normal architecture, triggering the development of liver fibrosis and, eventually, cirrhosis. The telomere-shortening effect of chronic liver injury can synergize with inherited genetic variants in the TERT and TERC genes that result in decreased activity of the telomerase complex to accelerate the premature development of liver fibrosis and cirrhosis ^20, 21^. Because cirrhosis is a precursor to HCC, the telomere hypothesis holds that this telomere shortening results in chromosomal instability that drives cancer initiation. Stabilization of the telomeric DNA through either increased telomerase expression or alternative mechanisms of telomerase activation is a key mechanism of cellular immortalization, allowing cells to survive and proliferate indefinitely ^22^. Mutations in the TERT promoter region have now been shown to be the most common mutation in HCC and the most frequent mechanism of telomerase activation. The mutations result in the formation of novel ETS transcription factor-binding sites upstream of the TERT start site, which leads to increased TERT transcript expression. Mutations in the TERT promoter region occur in 30–60% of HCCs ^23– 26^. Nault *et al.* found TERT promoter mutations not only in 59% of HCCs but also in 25% of cirrhotic preneoplastic lesions, suggesting that this is likely a driver mutation ^24^. Interestingly, TERT promoter mutations are conspicuously less common in HBV-induced HCCs, but these tumors have been shown to have recurrent integrations of HBV sequences into the TERT gene locus, which serves as a complementary mechanism for telomerase activation ^27– 29^.

***TP53 pathway mutations.*** TP53 is a widely recognized tumor suppressor, and low p53 levels or mutations in p53 are found in multiple cancer types. Wild-type p53 promotes apoptosis and cell cycle arrest, therefore, inactivating mutations in the p53 gene, or other pathway components, may render hepatocytes susceptible to the effects of other carcinogens that activate oncogenic pathways and may also predispose to the development of HCCs with a more aggressive phenotype ^30^. The frequency of p53 gene mutation in HCCs ranges from 18% to 50%, depending on the underlying etiology. Consequently the rate of p53 mutations varies in different geographic regions, reflecting the regional variations in HCC etiology ^26, 29, 31– 33^. In particular, dietary exposure to fungal aflatoxin (AFB1) results in a specific p53 mutation most commonly reported at codon 249; this is considered to be a driver mutation since it is also found in the normal livers of patients exposed to AFB1 ^34^. There is strong epidemiologic synergism between aflatoxin exposure and chronic HBV infection in the induction of HCC, and it has been shown that in patients infected with hepatitis B, expression of hepatitis B X (HBx) is associated with an approximately twofold increase in the incidence of G/C-to-T/A transversion mutations following AFB1 exposure ^35^. Other genes in the p53 pathway that are recurrently mutated in HCC include ATM and RPS6KA3 ^29^.

***Other common somatic mutations and hepatitis B virus integrations in hepatocellular carcinoma.*** Some of the other common mutations in HCC involve the Wnt/β-catenin pathway, including mutations in the β-catenin (CTNNB1) (18–40%), AXIN1 and AXIN2 genes ^31– 33^. These, along with other alterations in Wnt/β-catenin pathway components, are discussed below. Additional recently identified mutations in HCCs involve members of the chromatin remodeling pathway (ARID1A and ARID2) and the Janus kinase (JAK)/signal transducers and activators of transcription (STAT) pathway (JAK1, IL6R, and IL6ST), genes involved in ubiquitination (KEAP1), genes involved in RAS/MAPK signaling (RPS6KA3) and genes in the oxidative stress pathway (NFE2L2) ^36^.

HBV is known to recurrently integrate into the host genome and promote hepatocarcinogenesis ^37^. Several studies of the sites of HBV integrations in the hepatocyte genome have identified genes recurrently targeted by HBV integration, including TERT, MLL4, RARβ, CCNE1, Cyclin A2, FN1, ROCK1, SENP5, ANGPT1, platelet-derived growth factor (PDGF) receptor, calcium signaling-related genes, ribosomal protein genes, epidermal growth factor receptor (EGFR), and mevalonate kinase carboxypeptidase ^28, 37– 40^. Several of these integrations are proposed to have direct or indirect pathogenic roles in the development for HCC. For example, MLL4 encodes a histone methyltransferase that plays an important role in epigenetic modification of gene expression ^41^. Also, HBV integration into specific genes has been noted to alter gene expression; for example, Sung *et al.* reported that samples with HBV integration had significantly higher expression of the TERT, MLL4, and CCNE1 genes than tumors not harboring HBV DNA integrations in these genomic regions ^39^. Moreover, HBV viral integration also results in deletions or translocations of the host genome and ultimately increases chromosomal instability, which also predisposes to cancer initiation ^42– 44^.

### Copy number variations and gene rearrangements

Copy number variations are structural alterations of the genome in which small or large segments of the chromosome are either amplified (gain of genomic DNA) or deleted (loss of genomic DNA). Such structural variations promote carcinogenesis by increased expression or activation of oncogenes and decreased expression or inactivation of tumor suppressors. A recent study of 125 HCCs reported focal amplifications in 32% of the HCCs, identifying CCND1 and FGF19 as genes recurrently amplified in HCC ^32^. Focal deletions appeared to be even more prevalent, being present in 40% of HCCs; deletions commonly involved the CDKN2A (encoding the p16 tumor suppressor), CDKN2B, AXIN1, and IRF2 genes ^32^. Another study, of 286 HCC patients, identified 29 recurrently amplified regions and 22 recurrently deleted regions with a high level of copy number changes. Genes commonly involved with copy number variations included CCND1, MET, CDKN2A, and CDKN2B ^45^. Other studies of smaller sample cohorts similarly reported regions exhibiting significant copy number variations and used this analysis as a strategy for identifying potential HCC driver genes ^46, 47^. Several of these alterations have known associated pathogenic mechanisms; for example, a proportion of HCCs activate TERT by focal amplification in the TERT region, and deletion of AXIN1 is one of the mechanisms mediating Wnt/β-catenin pathway activation in HCC.

Another form of somatic variation contributing to carcinogenesis is chromosomal rearrangements, which can result in the fusion of two genes by chromosome translocation, inversion, or deletion. In a recent breakthrough study, such a gene fusion was described in fibrolamellar HCC. This is a variant of HCC that arises in non-cirrhotic livers, usually in young persons, and has a distinct morphology. A chromosomal rearrangement involving an approximately 400-kilobase deletion in chromosome 19 results in the formation of a chimeric RNA encoding a protein containing the amino-terminal domain of DNAJB1 (a homolog of the molecular chaperone DNAJ), fused in frame with PRKACA (the catalytic domain of protein kinase A). This fusion appears to be highly specific for fibrolamellar HCCs, being identified in 100% of fibrolamellar HCCs, suggesting that this genetic alteration likely contributes to tumorigenesis and the unique morphology of this HCC subtype ^14, 15^.

### Epigenetic modifications

Epigenetics is defined by the presence of heritable states of gene expression without alteration in DNA sequences. Deregulated epigenetics contributes to carcinogenesis by influencing multiple mechanisms, including gene transcription, chromosomal stability, and cell differentiation. Epigenetic mechanisms include changes in the methylation, hydroxymethylation ^48^, or acetylation (or a combination of these) of particular DNA regions or of the histone proteins around which DNA is organized, as well as mechanisms of gene regulation by non-coding RNAs.

***DNA methylation.*** Dysregulated methylation targets multiple gene regions in HCC and is characterized by global and site-specific hypomethylation as well as site-specific hypermethylation. Global hypomethylation in liver cancer affects the structural-nuclear function by promoting chromosomal and genetic instability, whereas regional hypermethylation is often associated with silencing of tumor suppressor genes ^49^. Etiological factors such as chronic HBV and HCV infection may cause dysregulated methylation during liver carcinogenesis ^50, 51^. Deng *et al.* reported on a subset of 15 genes that were found to be preferentially methylated in HCV-related HCC; the methylated genes belong to signaling pathways such as RAS/RAF/ERK and the Wnt/β-catenin pathways ^52^. Methylation of the GSTP1 and E-cadherin promoters has been reported to preferentially occur in hepatitis B-related HCC ^53^. Another epigenetic mechanism for tumorigenesis by HBV is targeted deregulation of DNA methyltransferases (DNMTs) by HBx, which promotes both specific regional hypermethylation and global hypomethylation ^50^.

CDKNA2 promoter hypermethylation leading to suppression of p16 is a commonly observed event in HCC ^54, 55^. P16 is a cell cycle regulator and a tumor suppressor; hence, its suppression promotes tumor progression. Other commonly methylated genes in HCC include RASSF1A ^56^, GSTP1 ^57, 58^, SOCS-3 ^59^, and MGMT ^60^. More recently, whole genome approaches to characterizing changes in methylation have resulted in more comprehensive assessments of gene methylation in cancer and allowed integration of whole genome methylation with whole genome gene expression data, identifying genes whose expression is truly modulated by methylation ^61– 63^.

***Histone modification.*** Histones regulate gene expression by determining the open or closed state of chromatin; thus, the level of gene expression depends on the post-translational modifications of histones in the transcriptional unit. Post-translational histone modifications such as acetylation and methylation of lysine and arginine residues, phosphorylation of serine and threonine residues, and ubiquitination of lysines are directed at the histone tails that protrude from the nucleosomes. The role of such DNA-protein modifications in liver carcinogenesis and HCC progression is not fully understood. High levels of trimethylated histone H3 lysine 4 (H3K4me27) have been shown to correlate with reduced overall survival and poor prognosis in HCC ^64^. In another study, high levels of H3K27me3 correlated with aggressive tumor features such as vascular invasion, large tumor size, multiplicity of tumors, and poor differentiation, and predicted worse prognosis in HCC ^65^. The regulation of histone modifications appears to be specific to different etiologies. For example, in patients with HBV, the oncogenic HBx protein can interact directly with the CBP/p300 histone acetyl-transferase complex, thus altering gene transcription and promoting tumorigenesis ^66^.

***Chromatin remodeling.*** Chromatin remodeling describes the process of dynamic changes in chromatin structure that regulate gene expression, apoptosis, and DNA repair. Disruption in chromatin remodeling can contribute to cancer initiation and progression. Awareness of the influence of chromatin remodeling processes in HCC development and growth is increasing. For example, the switch/sucrose non-fermenting (SWI/SNF) complex is a multi-protein complex essential for chromatin remodeling. SWI/SNF comprises dozens of proteins, including SMARCB1 and SMARCA4, and plays a key role in epigenetic regulation of gene expression. Recent studies have described frequent mutations in SW1/SNF chromatin remodeling complex genes such as ARID1A, ARID1B, and ARID2 in HCC ^67^. ARID2, which encodes a SW1/SNF regulatory subunit protein, was found to be mutated in 18.5% of HCV-related HCCs ^68^. Most ARID1A and ARID2 mutations detected in cancer cells to date are inactivating mutations, suggesting that both proteins function as tumor suppressors.

The polycomb group of chromatin remodeling proteins also plays a role in heritable gene silencing. There are two polycomb repressive complexes, denoted PRC1 and PRC2. The mechanisms by which they repress gene expression are incompletely understood, but PRC1 is believed to work through ubiquitin ligases which covalently modify histone tails, whereas the main function of PRC2 is to methylate histone H3K27 ^69, 70^. EZH2, a subunit of PRC2, is a methyltransferase that mediates gene silencing ^71^. EZH2 mRNA transcript and protein levels are consistently elevated in HCC in comparison with non-tumor liver tissues, and high levels of EZH2 are associated with HCC invasion and metastasis and poor prognosis ^72, 73^. EZH2 has been shown to promote hepatocarcinogenesis by silencing Wnt antagonists and consequently activating Wnt/β-catenin signaling ^72^. Knockdown of EZH2 in liver cancer cell lines also reduces levels of the repressive H3K27me3 histone, resulting in re-expression of a distinct subpopulation of tumor suppressor miRNAs that control cell motility and adhesion ^74^.

***MicroRNAs.*** MicroRNAs (miRNAs) are small non-coding RNAs that regulate the translation of many genes. They have emerged as key factors regulating multiple biological processes, including development, differentiation, and cell proliferation. MiRNAs mediate carcinogenesis and progression of HCC by directly or indirectly controlling the expression of key proteins involved in cancer-associated pathways.

Chronic hepatitis and hepatocarcinogenesis are associated with profound changes in miRNA expression ^75^. MiR-155, a positive regulator of liver inflammation, is upregulated in both serum and monocytes of patients with chronic HCV. The HCV core, NS3, and NS5 proteins and the HCV-induced TLR4 and TLR8 ligands all mediate increased miR-155 and tumor necrosis factor alpha (TNFα) production in chronic HCV infection, and this in turn promotes hepatocarcinogenesis by activating Wnt signaling ^76^. MiR-122, one of the most abundant liver-specific miRNAs, is downregulated in about 70% of HCCs ^77^. MiR-122 acts as a tumor suppressor, inducing apoptosis of HCC cells by directly targeting the Wnt/β-catenin pathway. Suppression of miR-122 is associated with intrahepatic metastases and tumor recurrence after surgical resection ^78– 80^. In a phenomenon reflecting the complexity of cancer genetic mechanisms, deletions in miR-122a have been shown to promote the epithelial-mesenchymal transition (EMT) and spontaneous HCC formation in mouse models ^81^. Other miRNAs such as miR-224, miR-224-3p, and their precursors are upregulated in HCV-associated cirrhosis, HCV-associated HCC, and HBV-associated liver failure compared with normal liver tissue ^82^. Apoptosis inhibitor 5 (API-5) and SMAD4 have been identified as target genes for miR-224, and expression of miR-224 is associated with poor survival ^83^. Overexpression of miR-224 increases SMAD4 protein in murine granulosa cells without increasing SMAD4 RNA levels, suggesting a post-transcriptional role for miR-224 ^83^. Tumor suppressive miRNAs, including miR-1, miR-124, miR-214, miR-34-A, and miR-449, target mRNAs involved in cell growth, metastasis, or suppression of apoptosis and usually are downregulated in HCC. In contrast, oncogenic miRNAs, including miR-221, miR-224, miR-21, and miR210, promote tumor progression and are upregulated in HCCs ^84^. There are active efforts under way to exploit modulation of miRNA levels for HCC therapy ^85^.

***Long non-coding RNAs.*** Long non-coding RNAs (lncRNAs) are another class of transcribed RNAs that do not encode proteins. LncRNAs regulate gene expression and protein synthesis by diverse mechanisms. Aberrant expression of lncRNA can affect genes involved in hepatocarcinogenesis, microvascular invasion, and metastasis. Most lncRNAs are undetectable or expressed at low levels in normal liver tissue but upregulated in HCCs. Alterations in several lncRNAs have been described in HCC. In HBV-related HCC, HBx induces upregulation of an lncRNA known as highly upregulated in liver cancer (HULC), which in turn suppresses the expression of p18 and facilitates proliferation of HCC ^86^. Depletion of HULC results in significant deregulation of several genes involved in liver cancer, and higher HULC levels are observed in the plasma of patients with higher histological grades of HCC or positive HBV status ^87^. HULC is specifically increased in blood and tumor tissues of patients with HCC and may have utility as a biomarker for HCC. Another lncRNA, designated lncRNA-HEIH, has also been shown to be overexpressed in HCC ^88^. Downregulation of lncRNA-HEIH induced G _0_/G _1_ cell cycle arrest. The level of overexpression of lncRNA-HEIH was associated with recurrence of tumor in HBV-related HCC and was also an independent risk factor for survival ^88^. Lnc-RNA-Dreh, also known as the HBx-related lncRNA, acts as a tumor suppressor by targeting the intermediate filament protein vimentin in HBV-associated HCC, leading to inhibition of growth and metastases both *in vitro* and *in vivo* ^89^. The novel lncRNA-associated microvascular invasion in HCC (lnc-MVIH) has been found to promote tumor growth and intrahepatic metastasis through activation of angiogenesis ^90^. It has been shown that lnc-MVIH inhibits secretion of phosphoglycerate kinase 1 (PGK1), a glycolytic enzyme known to inhibit angiogenesis ^90, 91^. The lncRNA HOX transcript antisense RNA (HOTAIR), which can reprogram chromatin to promote cancer metastasis, has been found to be overexpressed in HCC, and patients with HOTAIR overexpression had significantly poorer prognosis and higher recurrence rates than those with low HOTAIR expression ^92, 93^. Mineral dust-induced gene (MDIG), an lncRNA regulated by the c-myc oncogene, was also found to be overexpressed in HCC. MDIG expression was noted in the nuclei of neoplastic cells and had higher expression in larger and poorly differentiated HCCs ^94^.

Another mechanism by which lncRNAs induce hepatocarcinogenesis was recently described by Lau *et al.*, who demonstrated that HBV viral integrations into the host genome can result in viral-human chimeras which function as lncRNAs and can promote tumorigenicity ^95^. An HBV integration in chromosome 8 resulted in co-transcription of long-interspersed nuclear element 1 (LINE1), which is usually silent in the human genome, with the HBx gene of HBV. They were further able to demonstrate that the HBx-LINE1 chimera was functionally relevant as it led to Wnt/β-catenin pathway activation and resulted in tumor progression. Other recently described lncRNAs which play a role in hepatocarcinogenesis and also have the potential to serve as biomarkers include MALAT ^96^, HOTTIP ^97^, and MEG3 ^98^.

## Key signaling pathways in liver carcinogenesis

Alterations in numerous signaling pathways occur in cancer, and several specific pathways have been observed to be dysregulated in HCC. Changes in liver tissues induced either by chronic viral infection or by exposure to hepatotoxic agents cause upregulation of components of a number of cellular signaling pathways. The predominant pathways involved in HCC pathogenesis include pathways regulating growth factor signaling such as the insulin-like growth factor (IGF), epidermal growth factor (EGF), PDGF, fibroblast growth factor (FGF) and hepatocyte growth factor (HGF/MET); pathways related to cell differentiation such as the WNT, Hedgehog, and Notch pathways; and pathways related to angiogenesis such as the vascular endothelial growth factor (VEGF) and FGF pathways. The major signaling mediators downstream of the receptor tyrosine kinases are the Ras/Raf/MEK/ERK and P13K/AKT/mTOR cascades ^99^. There are also substantial contributions to liver carcinogenesis from pathways regulating the tumor microenvironment and pathways that disrupt anti-tumor immunity ^88, 100^. Below, we describe the most commonly altered pathways and the mechanisms that lead to specific pathway activation. Besides improving our understanding of the pathogenesis of HCC, this information is valuable for the identification of novel drug targets.

### WNT/β-catenin signaling pathway

The WNT/β-catenin signaling pathway is implicated in embryogenesis, differentiation, cell proliferation, and tumorigenesis and is one of the most commonly disrupted pathways in HCC ^101^. Nineteen Wnt ligands have been described and there are 10 transmembrane frizzled receptors to which they bind, leading to either canonical (β-catenin-dependent) or non-canonical (β-catenin-independent) Wnt pathway activation. Activation of the canonical Wnt signaling pathway results in accumulation of β-catenin in the cytoplasm and translocation to the nucleus, where it binds to transcription factor TCF/LEF and activates downstream target genes. Gene mutations that activate WNT/β-catenin signaling are seen in up to 50% of HCCs. The most common are activating mutations in CTNNB1, which result in stabilization of β-catenin ^32^. Additionally, mutations in AXIN1 (in 3–16% of HCCs) and AXIN 2 (in about 3% of HCCs) which are both negative regulators of the Wnt pathway, as well as inactivation of the tumor suppressor gene adenomatous polyposis coli (APC), contribute to Wnt pathway activation ^32^. There are multiple additional mechanisms of Wnt pathway activation. In fact, those HCCs that exhibit Wnt/β-catenin pathway activation without CTNNB1 mutation appear to be distinct from those with CTNNB1 mutations. HCCs with Wnt/β-catenin pathway activation without CTNNB1 mutation are preferentially seen in HBV-infected patients and are associated with high chromosomal instability and an aggressive phenotype ^102^. In contrast, HCCs with CTNNB1 mutation are usually low-grade tumors with good prognosis ^103, 104^.

There are several other mechanisms for activation of Wnt signaling in HCCs apart from mutations involving the pathway. A subset of HCCs with Wnt pathway activation in the absence of CTNNB1 mutation show evidence of crosstalk with the transforming growth factor-beta (TGFβ) pathway ^102^. Overexpression of Wnt ligands ^105^ or frizzled receptors ^106^ and epigenetic changes in secreted frizzled-related protein-1 ^107^ are other mechanisms by which the Wnt pathway is activated in HCC.

### Receptor tyrosine kinase pathways

Activation of receptor tyrosine kinases induce the Ras-mitogen-activated protein kinase (MAPK or extracellular signaling regulated kinase, or ERK) and phosphatidylinositol 3-kinase (PI3K)-Akt kinase signaling pathways in about 50% of HCCs ^108^. Ligand binding and phosphorylation of several growth factor tyrosine kinase receptors, including the EGFR, FGFR, HGFR/c-MET, the stem cell growth factor receptor (c-kit), and VEGFR, lead to activation of the MAPK and PI3K pathways. Ras/Raf/MEK/ERK (MAPK) pathway activation in turn activates proto-oncogene cFos and transcription factor AP-1/c-Jun, which induce transcription of genes that drive cell proliferation ^109^. Activation of the PI3K-Akt kinase signaling pathway through the insulin or IGF receptors (such as IGFR1) results in disruption of the mammalian target of rapamycin (mTOR) pathway, which occurs in about 40% to 50% of cases of HCC, thus promoting carcinogenesis ^110^. This pathway can also be dysregulated by constitutive activation of PI3K because of loss of function of the tumor suppressor gene PTEN by either mutation or epigenetic silencing. Sorafenib, which is currently the only approved therapy for advanced HCC, acts in part by blocking the RAS/MEK/ERK pathway ^111^. Although the role of EGFR mutations in HCC pathogenesis is small, the EGFR pathway appears to play a significant role in HCC initiation, as a polymorphism in the EGF gene (SNP rs44449030) (G/G versus A/A) was associated with a fourfold increased risk for HCC in patients with cirrhosis ^112^. Another receptor tyrosine kinase pathway that has garnered increased attention recently is the HGF-MET pathway. Expression of a MET gene signature was associated with vascular invasion and poor prognosis in human HCC, and in a subgroup analysis of the Sorafenib HCC Assessment Randomized Protocol (SHARP) trial, high plasma HGF levels were found to correlate with poor survival in patients who received sorafenib ^113, 114^. The FGF family consists of 23 members whose multiple ligands interact with four FGF receptors (FGFR1-4), of which FGFR4 is the most abundant receptor expressed in hepatocytes. The ligand FGF19 binds to FGFR4 and regulates bile acid synthesis and hepatocyte proliferation. Recent data have shown that FGF19-FGFR4 pathway activation may play a key role in a proportion of HCCs and this pathway is a potential therapeutic target ^115, 116^. A new small molecule-specific inhibitor of FGFR4 has been shown to be efficacious against HCCs with an intact FGF pathway ^117^.

A number of receptor tyrosine kinases use heparan sulfate as a co-receptor, and heparan sulfate on the cell surface or in the extracellular matrix can also serve as a storage or concentration site for heparan sulfate-binding ligands. A pair of heparan sulfate sulfatases, SULF1 and SULF2, have been shown to modulate HCC carcinogenesis and tumorigenesis through effects on the affinity of heparan sulfate for heparan sulfate-binding receptor tyrosine ligands. Besides the efforts to identify specific inhibitors of receptor tyrosine kinases, there are efforts under way to develop sulfatase inhibitors as potential anti-cancer agents ^118– 120^.

### Vascular endothelial growth factor and other angiogenesis pathways

HCC is a highly vascular tumor and angioneogenesis is a dominant feature of this tumor, with the hepatic artery as the major source of its blood supply. VEGF and angiopoetins play a prominent role in promoting and sustaining neoangiogenesis in HCC ^121, 122^. These principles have been used for developing effective therapeutic strategies against HCC, such as transarterial chemoembolization (TACE), which works by blocking the arterial supply to the tumor, and sorafenib, which inhibits the angiogenic effects of growth factors such as VEGF. In spite of the rich vascular supply, hypoxia is present in focal areas of the tumor because of disorganized capillarization and the presence of leaky, immature vessels ^123^. Hypoxia in HCC, in turn, leads to induction of growth factors such as hypoxia-inducible factors 1 and 2 (HIF 1 and 2) and IGFs that promote further tumor angiogenesis by transcriptional activation of hypoxia-responsive genes and lead to tumor progression and metastases ^124, 125^. HIFs have also been shown to impart chemo- and radio-resistance to HCC tumors, leading to failure of transarterial therapies ^126^, and hence their overexpression is associated with poor prognosis.

### Transforming growth factor-beta pathway

The TGFβ pathway has long been recognized to play a dual role in cancer: it has the ability to suppress cellular growth in the early stages of cancer initiation and the paradoxical ability to promote invasiveness and angiogenesis in later stages ^127^. With this in mind, Coulouarn *et al.* used transcriptome analysis to identify early and late TGFβ signatures in HCC and showed that the late TGFβ signature was associated with shortened survival time compared with patients with the early TGFβ signature. Also, tumors expressing late TGFβ-responsive genes displayed an invasive phenotype and increased tumor recurrence ^128^. The poor prognosis observed is likely explained by the observation that, in late stages of HCC, TGFβ is known to promote EMT, which is a key mechanism involved in promoting tumor metastases ^129^. In another widely recognized transcriptomic classification of HCCs, the subgroup S1 was noted to be associated with WNT pathway activation which was the result of TGFβ activation ^102^. These data suggest that TGFβ pathway activation is likely involved in a significant subset of HCCs and hence is a rational drug target ^130^. In recent work from our group, we have shown that the heparan sulfate sulfatases promote HCC tumor progression by activating the TGFβ pathway ^118, 131^.

### JAK/STAT pathway

STATs are activated by a variety of cytokines, hormones, and growth factors. The activation occurs through tyrosine phosphorylation by JAKs. Activated STATs stimulate the transcription of suppressors of cytokine signaling (SOCS) genes that, in turn, bind to phosphorylated JAKs and their receptors to inhibit this pathway, thus preventing over-activation of cytokine-stimulated cells. Therefore, SOCS are a part of a negative feedback loop in the JAK/STAT pathway. JAK stimulation of STATs activates cell proliferation, migration, differentiation, and apoptosis, and deregulation of the inhibitors leads to human diseases, including cancer. Inactivation of the JAK-binding proteins SOCS1 and SSI-1 and activation of the JAK/SKAT pathway have been reported in HCC ^132, 133^.

### Ubiquitin proteasome pathway

The ubiquitin proteasome system is involved in cellular protein degradation. After being tagged with ubiquitin, cellular proteins are degraded by the proteasome. The ubiquitin-activating enzyme E1 mediates ATP-dependent transfer of ubiquitin to a ubiquitin-conjugating enzyme (E2), which in turn transfers the ubiquitin either directly to the substrate protein or to a downstream ubiquitin ligase E3, which then ubiquitinates the specific substrate or substrates ^109^. Several cancer-related proteins such as the tumor suppressors p53, p27, pRb, PTEN, the EGF receptor tyrosine kinase, TGFβ, and other cell cycle regulators and oncogenic molecules are regulated by the ubiquitin-proteasome system ^134^. E3 ligases that have been shown to function as tumor suppressors in HCC include the mouse double minute 2 (MDM2) and BRCA1, which target p53 for ubiquitination and degradation ^135^; Smad ubiquitinization regulatory factor-2 (Smurf-2), which targets Smad proteins and the TGFβ receptor complex for degradation ^136^; and the FRA6E fragile site protein Parkin, which targets cyclin E and P38 ^137^.

### Immune escape mechanisms in hepatocellular carcinoma

The liver faces a constant stream of exogenous antigens from the gut reaching it via the portal vein and hence has a unique tolerogenic immune environment. HCC tumor cells developing in this background are able to evade immune surveillance by several mechanisms. Regulatory T cells (Tregs) are a subset of CD4 ^+^ T cells that suppress effector CD8 T cells, thus playing an important negative role in anti-tumor immunity ^138^. Several studies have shown that HCC tumor tissues appear to be infiltrated with Tregs and also that patients with HCC have an increased number of circulating Tregs, thus implying that they likely play a pathogenic role in HCC ^139^. Another mechanism of immune suppression in the tumor microenvironment is an increase in immunosuppressive cytokines—such as interleukin-4 (IL-4), IL-5, IL-8, and IL-10—with simultaneous suppression of immune activating cytokines: IL-1, TNF, and interferon gamma ^140^. This unique cytokine signature has been shown to promote tumor metastases, and circulating levels of IL-10 were reported to be associated with poor prognosis ^140– 142^. An alternate mechanism of immune evasion that has gained recent attention is modulation of tumor immunity by the programmed cell death-1 (PD-L1/PD-1) immune checkpoint pathway ^143, 144^. Increased expression of PD-L1 in tumor cells induces apoptosis of effector T cells and contributes to immune evasion ^144^. Immune checkpoint inhibitors are being increasingly recognized as effective therapeutic option for cancers like melanoma, and a recent early report from a phase I/II study has suggested that, in patients with advanced HCC, the anti-PD-1 monoclonal antibody nivolumab has clinical efficacy without significant toxicity ^145^.

### Cancer stem cells in hepatocellular carcinoma

The origin of self-renewing cells in HCCs is not clearly understood, and, recently, growing evidence supports the novel notion that tumor initiation is likely driven by a subset of cells with stem cell features. These cancer stem cells (CSCs) are considered to be responsible not just for tumor initiation but also for tumor persistence, relapse, and metastasis, thus leading to a more aggressive tumor phenotype ^146^. CSCs also render a tumor chemoresistant and radioresistant, which may explain why HCCs are generally resistant to conventional chemotherapies and also why newer-generation therapies like sorafenib, which do not target CSCs, are associated with frequent tumor relapse after therapy. Hence, identification and characterization of signaling pathways and biomarkers associated with CSCs are priorities for developing new paradigms of molecular cancer therapeutics in the treatment of HCC ^147^. CD133 antigen is considered to be a marker for CSCs, and HCCs with high CD133 expression were associated with poor survival and high recurrence rate ^148^. One of the mechanisms identified in the induction of stemness in HCCs is IL-6-mediated activation of STAT3, which in turn leads to transcriptional activation of CD133 ^149^. Another study described CD24 to be a functional marker of liver tumor-initiating cells that drives tumorigenesis through STAT3-mediated regulation of a self-renewing gene NANOG ^150^. Further understanding of the role of CSCs in the pathogenesis of HCC will hopefully unveil new therapeutic targets.

## Conclusions and future directions

**Figure 2.**
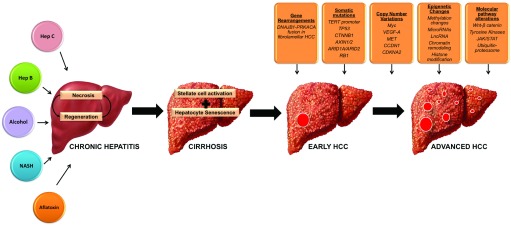
Pathogenesis of Hepatocellular Carcinoma. Chronic exposure of the liver to injury from viral hepatitis, alcohol abuse or NASH causes repeated hepatocyte damage and sets up a vicious cycle of cell death and regeneration which eventually results in cirrhosis. The resultant genomic instability leads to initiation of HCC. Step wise accumulation of multiple genetic events including gene rearrangements, somatic mutations, copy number alterations, epigenetic changes and growth factor pathway alterations eventually lead to tumor progression and metastases. Abbreviations: Hep B, hepatitis B; Hep C, hepatitis C; HCC, hepatocellular carcinoma; NASH, non-alcoholic steatohepatitis.

HCC is a heterogeneous malignancy resulting from diverse causes of chronic liver injury, with viral hepatitis being the most common etiology. Regardless of the etiology, there appears to be a final common pathway in the pathogenesis of HCC in which repeated hepatocyte damage sets up a vicious cycle of cell death and regeneration which eventually results in genomic instability and initiation of HCC ( Figure 2). Recent advances in next-generation sequencing are playing a pivotal role in providing a more comprehensive understanding of the genomic landscape of HCC and in identifying driver mutations ( Table 1). Also, recognition of specific molecular pathways commonly involved in HCC initiation and progression is facilitating recognition of novel drug targets for HCC. Currently, sorafenib is the only systemic therapy approved for the management of advanced HCC. A deeper understanding of the molecular pathogenesis of HCC will be instrumental for new drug discovery, which is desperately needed for the thousands of patients with this lethal malignancy.

**Table 1.** Major molecular events in the pathogenesis of hepatocellular carcinoma.

**Genomic alterations**	Gene mutations	TERT promoter ^23– 26^ TP53 ^26, 29, 31– 33^ CTNNB1 ^31– 33^ AXIN1, AXIN2 ^31– 33^ ATM ^29^ RPS6KA3 ^29^ JAK1, IL6R, IL6ST ^36^ ARID1, ARID2 ^67, 68^
	Gene amplification/ Deletions	CCND1 ^32^ FGF19 ^32^ CDKNA2A, CDKNA2B ^32, 45^ AXIN1 ^32^ IRF2 ^32^ MET ^45^
**Epigenetic modifications**	DNA Methylation	GSTP1 ^53, 57, 58^ E-Cadherin ^53^ CDKNA2 ^54, 55^ RASSF1A ^56^ SOCS-3 ^59^ MGMT ^60^
	MicroRNA	MiR-155 ^76^ MiR-122 ^78– 80^ MiR-224 ^82– 84^ MiR-1 ^84^ MiR-224 ^84^ MiR-21 ^84^
	Lnc RNA	HULC ^86, 87^ HEIH ^88^ Dreh ^89^ MVIH ^90, 91^ HOTAIR ^92, 93^ MDIG ^94^ LINE1 ^95^
**Growth factor pathway** **alterations**	Major signaling pathways	Wnt/β –catenin ^101, 102^ Tyrosine kinase pathways- *EGF* ^112^, *HGF/c-MET* ^113, 114^, *FGF* ^115, 116^ *VEGF* ^121, 122^ IGF ^124, 125^ HIF 1,2 ^126^ TGF *β* ^128, 129^ Hedgehog

There are several challenges to applying the knowledge gained from understanding the molecular pathogenesis of HCC in the care of patients diagnosed with this malignancy. The significant epidemiologic and molecular heterogeneity of HCC has to be overcome before individualized recommendations can be derived from broad generalizations. With further discovery of molecular subclasses, we can hopefully identify more homogenous subgroups that can be specifically targeted for drug development. Also, there is an urgent need to identify and validate biomarkers that can be used for early, non-invasive diagnosis and for prognostication. The most critical need of the hour is recognition of druggable molecular targets that can promote drug discovery efforts. Further endeavors to create better experimental models, such as patient-derived xenografts, will encourage personalized drug discovery.
